# Multiplex SNaPshot—a new simple and efficient *CYP2D6* and *ADRB1* genotyping method

**DOI:** 10.1186/s40246-016-0073-3

**Published:** 2016-04-23

**Authors:** Songtao Ben, Rhonda M. Cooper-DeHoff, Hanna K. Flaten, Oghenero Evero, Tracey M. Ferrara, Richard A. Spritz, Andrew A. Monte

**Affiliations:** Human Medical Genetics Program, University of Colorado, Aurora, CO 80045 USA; Center for Pharmacogenomics, College of Pharmacy, University of Florida, Gainesville, FL 32610 USA; Department of Emergency Medicine, School of Medicine, University of Colorado, 12401 E. 17th Ave, B215, Aurora, CO 80045 USA; Rocky Mountain Poison & Drug Center, Denver Health and Hospital Authority, Denver, CO 80203 USA

**Keywords:** *ADRB1*, *CYP2D6*, Copy number variation, Gene deletion, Genotyping, Personalized medicine, Precision medicine

## Abstract

**Background:**

Reliable, inexpensive, high-throughput genotyping methods are required for clinical trials. Traditional assays require numerous enzyme digestions or are too expensive for large sample volumes. Our objective was to develop an inexpensive, efficient, and reliable assay for *CYP2D6* and *ADRB1* accounting for numerous polymorphisms including gene duplications.

**Materials and methods:**

We utilized the multiplex SNaPshot® custom genotype method to genotype *CYP2D6* and *ADRB1*. We compared the method to reference standards genotyped using the Taqman Copy Number Variant Assay followed by pyrosequencing quantification and determined assigned genotype concordance.

**Results:**

We genotyped 119 subjects. Seven (5.9 %) were found to be *CYP2D6* poor metabolizers (PMs), 18 (15.1 %) intermediate metabolizers (IMs), 89 (74.8 %) extensive metabolizers (EMs), and 5 (4.2 %) ultra-rapid metabolizers (UMs). We genotyped two variants in the β1-adrenoreceptor, rs1801253 (Gly389Arg) and rs1801252 (Ser49Gly). The Gly389Arg genotype is Gly/Gly 18 (15.1 %), Gly/Arg 58 (48.7 %), and Arg/Arg 43 (36.1 %). The Ser49Gly genotype is Ser/Ser 82 (68.9 %), Ser/Gly 32 (26.9), and Gly/Gly 5 (4.2 %). The multiplex SNaPshot method was concordant with genotypes in reference samples.

**Conclusions:**

The multiplex SNaPshot method allows for specific and accurate detection of *CYP2D6* genotypes and *ADRB1* genotypes and haplotypes. This platform is simple and efficient and suited for high throughput.

## Background

Cytochrome P450 family 2 subfamily D member 6 (*CYP2D6*) is one of the most important drug-metabolizing enzymes expressed in humans. The enzyme metabolizes 20–30 % of all xenobiotics including numerous antidepressants, antipsychotics, anti-emetics, analgesics, and cardiovascular medications. The expression of *CYP2D6* is highly polymorphic; more than 70 allelic variants have been identified, and deletion or duplication of the gene leads to variable enzyme function in individual patients [1, 2]. The *CYP2D6* gene has been mapped to chromosome 22q13.1 and consists of nine exons with an open reading frame of 1491 base pairs coding for 497 amino acids [2–5]. Variability in enzyme expression has been associated with altered drug effectiveness and safety [2]. In fact, there are four functional genotype groups identified for the enzyme, poor metabolizers (PMs), intermediate metabolizers (IMs), extensive metabolizers (EMs), and ultra-rapid metabolizers (UMs) based upon the number and activity of the gene copies the patients express (Table 1) [1, 6]. Therefore, many researchers and clinicians have targeted genotyping *CYP2D6* in efforts to improve drug therapy.

**Table 1:** Predicted *CYP2D6* enzyme function based upon the activity score derived from SNV identification

Predicted CYP2D6 enzyme function	Activity score
Poor metabolizer (PM)	0
Intermediate metabolizer (IM)	0.5
Extensive metabolizer (EM)	1.0–2.0
Ultra-rapid metabolizer (UM)	>2.0

Some genes have partial functionality based upon in vitro enzyme activity for specific substrates. This table is adapted from Crews et al*.* and Gaedigk et al. [1, 6]

Expanding on previously published work [7–9], we sought to determine the effectiveness and safety of metoprolol succinate utilizing a systems biology approach, whereby we genotyped the drug-metabolizing enzyme *CYP2D6* as well as the drug target, β1-adrenoreceptor (*ADRB1*). Additionally, we captured demographic and clinical factors that are necessary to understand the potential for individualized therapy in patients taking metoprolol succinate. Metoprolol is a B1 selective antagonist, known as a beta-blocker. Beta-blockers are first-line treatment for heart failure, hypertension (HTN), angina, and myocardial infarction [10–14]. In 2011, 34.5 million prescriptions for metoprolol were written in the USA [15]. Thus, metoprolol is a first-line therapy for several of the most common chronic diseases and is one of the most commonly prescribed drugs in the USA. CYP2D6 is the only clinically pertinent pathway of metoprolol metabolism, and polymorphisms have been associated with altered levels of metoprolol [16, 17]. *ADRB1* is the drug target, and polymorphisms in this receptor have been associated with variable drug response [7, 18]. Prediction of drug response with knowledge of one without knowledge of the other could be incomplete since both contribute to the ultimate clinical effect [19]. This highlights the importance of accounting for both factors. Prior investigators have identified imperfect associations with *CYP2D6* [16, 17] and *ADRB1* [7, 18] genotypes, but no study has been appropriately powered to account for variability in both genotypes [19]. No single assay accounts for variants in both genes. Given that multiple single-nucleotide variants (SNVs) and the presence of gene duplication can affect *CYP2D6* and *ADRB1*, a simple, efficient, and inexpensive method that identifies both SNVs and gene duplication is required to allow for an adequately powered systems biology approach.

*CYP2D6* and *ADRB1* variants have been identified using long-range polymerase chain reaction (XL-PCR) with PCR restriction fragment length polymorphism (PCR-RFLP) or microarray analysis. Microarray analysis remains expensive for large-scale genotyping. Commercially available tests cost a minimum of $500 per patient. XL-PCR with PCR-RFLP accounts for allele variants, and multiplication is inexpensive, easy, and reliable in patients regardless of ethnicity or race [6]. However, XL-PCR with PCR-RFLP requires numerous enzymatic digestions and multiple amplification steps to identify more than one polymorphism in the *CYP2D6* gene. In order to conduct a large-scale clinical trial to determine the effectiveness and safety of metoprolol in treating hypertension, we sought to develop a simple, rapid, yet less expensive assay for *CYP2D6* and *ADRB1* that accounts for numerous polymorphisms including gene duplications. Drug effectiveness data will be presented at a later date when the trial closes.

## Material and methods

### Study design and setting

This was a prospective observational clinical trial (NCT02293096) that enrolled patients with uncontrolled HTN from clinics, the emergency department, and across the community at the University of Colorado Hospital in Aurora, Colorado.

### Subjects

In accordance with the University of Colorado IRB approval, we enrolled subjects with uncontrolled HTN between 30 and 80 years of age. Exclusion criteria included end-stage liver disease, glomerular filtration rate < 60 ml/min/1.73 m^2^, pregnancy, American Society of Anesthesiologists (ASA) classification of >3, prisoners or wards of the state, decisionally challenged, heart rate < 60 beats per minute, AV block > 240 ms, active reactive airway disease, illicit drug use in the preceding 30 days (excluding marijuana), allergy to metoprolol succinate, or severe peripheral arterial circulatory disorders.

### Drug intervention

Patients were initially managed according to the Eighth Joint National Committee guidelines for management of HTN [20]. Patients were started on angiotensin-converting enzyme inhibitor or angiotensin receptor blockers as first-line therapy. If blood pressure remained uncontrolled, then metoprolol succinate was added. Patients were then followed up and metoprolol titrated weekly for 4 weeks. Drug effectiveness data will be presented at a later date.

### DNA isolation

Genomic DNA was extracted from whole blood via the Puregene® Blood Core kit B (Qiagen) according to the manufacturer’s instructions.

### Long-range PCR analyses to determine CYP2D6 gene multiplication

We analyzed *CYP2D6* duplication using long-range PCR with the primers described by Lovlie et al. [21]. This primer combination amplifies a 5.2-kb PCR fragment from the *CYP2D7-CYP2D6* intergenic regions in all individuals and a 3.6-kb PCR fragment from the *CYP2D6-CYP2D6* region in individuals with a duplication of the gene [22]. The amplification was done using the Phusion High-Fidelity DNA Polymerase kit (New England Biolabs). The reaction components included 2 μl of 5× Phusion HF buffer, 0.2 μl of 25 mM dNTPs, 0.5 μl of 10 μM/μl forward primer, 0.5 μl of 10 μM/μl reverse primer, 1 μl of template DNA, 0.2 μl of Phusion DNA polymerase, 0.3 μl DMSO, 2.5 μl of BETAIN, and 2.8 μl of nuclease-free water. The total reaction volume was 10 μl. Thermal cycling conditions were as follows: 98 °C for 30 s followed by 35 cycles of 98 °C for 10 s, 67 °C for 30 s, and 72 °C for 2 min 30 s. After cycling, samples were stored at 4 °C. PCR products were analyzed by electrophoresis.

### Long-range PCR analyses to determine CYP2D6 gene deletion

To detect deletion of the entire *CYP2D6* gene, (CYP2D6 *5) we performed a multiplex longer-PCR reaction with the primers described by Okubo et al. [23]. The reaction **c**omponents included 2 μl of 5× Phusion HF buffer, 0.2 μl of 25 mM dNTPs, 0.2 μl of 10 μM F1 primer, 0.2 μl of 10 μM R2 primer, 2 μl of 10 μM F3 primer, 1 μl of template DNA, 0.2 μl of Phusion DNA polymerase, 2.5 μl of BETAIN, and 1.7 μl of nuclease-free water. The PCR conditions were as follows: initiation at 98 °C for 3 min, 35 cycles of 98 °C for 10 s and 70 °C for 30 s, termination at 72 °C for 2 min 30 s, and a final elongation at 72 °C for 10 min. The PCR products were examined by electrophoresis and include the amplification of a 3.5-kb fragment, which indicates CYP2D6 *5, and a 4.7-kb fragment, which indicates *CYP2D6* wild type.

### SNV selection

We detected 20 variants associated with altered *CYP2D6* enzyme activity. SNVs were chosen because they are representative of major haplotypes associated with altered enzyme function without providing redundancy of rsIDs leading to the same functional activity within the same allele variant (Table 2). If none of these SNVs were identified, the allele designation was defaulted to the reference allele, CYP2D6*1. Haplotype analysis for *CYP2D6* was based upon predicted enzyme activity of the SNVs identified in the genotyping stage.

**Table 2:** *CYP2D6* and *ADRB1* variant alleles with rsIDs and subsequent effect on the gene sequence

Allele	Major nucleotide variation	SNV	Effect
*1	Presumed	NA	Wild type
*2	2850C>G	rs16947	Arg296Cys
*3	2549delA	rs35742686	Frameshift
*4	100C>T	rs1065852	Pro34Ser
	1846G>A	s3892097	Splicing defect
*6	1707delT	rs5030655	Frameshift
*7	2935A>C	rs5030867	His324Pro
*9	2615_2617delAAG	rs5030656	Lys281del
*10	100C>T	rs1065852	Pro34Ser
*12	124G>A	rs5030862	Gly42Arg
*14	1758G>A	rs5030865	Gly169Arg
*17	1023C>T	rs28371706	Thr107Ile
	2850C>T	rs16947	Arg296Cys
*19	2539_2542delAACT	rs72549353	255Frameshift
*20	1973_1974insG	rs72549354	211Frameshift
*38	2587_2590delGACT	rs72549351	271Frameshift
*40	1863_1864insTTTCGCCCCX2	rs72549356	174_175insFRP × 2
*41	2850C>T	rs16947	Arg296Cys
	2988G>A	rs38371725	Splicing defect
*42	3259_3260insGT	rs72549346	363Frameshift
*49	100C>T	rs1065852	Pro34Ser
	1611T>A	rs1135822	Phe120Ile
*69	100C>T	rs1065852	Pro34Ser
	2850C>T	rs16947	Arg296Cys
	2988G>A	rs38371725	Splicing defect
ADRB 1 Ser49Gly	A>G	rs1801252	Ser49Gly
ADRB 1 Gly389Arg	G>C	rs1801253	Gly389Arg

Additionally, two *ADRB1* SNVs were genotyped (Table 2) because haplotypes of these alleles are known to be associated with altered clinical response to metoprolol treatment (Table 3). If neither of these SNVs were identified, the allele was defaulted to Ser49 and Gly389, the reference allele.

**Table 3:** ADRB1 haplotype and associated metoprolol clinical effect

*ADRB1* haplotypes	Metoprolol clinical effect
49Ser389Arg/49Ser389Arg	Threefold greater diastolic blood pressure reduction [7, 12]
49Ser389Arg/49Gly389Arg	Good responder [12]
49Ser389Gly/49Gly389Arg and 49Ser389Gly/49Ser389Gly	Non-responders [7, 12]

### Genotyping

Genotyping was performed using two separate multiplex reactions. First, we used PCR to amplify a purified template DNA fragment that included the target nucleotide. We designed a total of eight pairs of PCR primers (Table 4) that were divided into two separate pools. The PCR was performed with the AmpliTaq® Gold kit (Applied Biosystems) using a hot start/touchdown PCR assay. The 10 μl reaction included 1 μl of template DNA, 1.6 μl of 10× GeneAmp® PCR Buffer, 0.15 μl of 25 mM/each dNTPs, 0.4 μl of 1 μM/each primer mix, 2.15 μl of 50 mM MgCl_2_, 0.15 μl of 5 U/μl AmpliTaq Gold, and 4.55 μl of nuclease-free water. Thermal cycling conditions were as follows: 94 °C for 5 min followed by 15 cycles of 94 °C for 30 s; annealing temperature steps down every cycle by 0.5 °C (from 63 to 56.5 °C) every 30 s and then 72 °C for 1 min. The annealing temperature for the final 25 cycles was 56 °C with a denaturation temperature of 94 °C for 30 s and extension temperature of 72 °C for 1 min. After PCR, the products were first treated with shrimp alkaline phosphatase (SAP) and Exonuclease Ι (Exo Ι) to remove excess primers and dNTPs. Two units of SAP and 1 unit of Exo Ι were added to 5 μl of PCR product and incubated at 37 °C for 30 min and then 80 °C for 15 min. Second, we performed multiplex single-base extraction (SBE) reactions. We designed 19 SBE primers and divided the reactions into two pools. See Table 5. Each SBE reaction was carried out in a 10 μl final volume containing 5 μl of SNaPshot Multiplex Ready Reaction Mix, 2 μl of pooled PCR products, 1 μl of pooled SNaPshot primers (0.3 μmol/each μl), and 2 μl of deionized water. Extension was performed for 25 cycles under the following conditions: 96 °C for 10 s, 50 °C for 5 s, and 60 °C for 30 s. To remove ddNTPs, the SBE reactions were then treated with 1.0 unit of calf-intestinal phosphatase (CIP) and incubated at 37 °C for 30 min. The enzyme was deactivated by incubating at 80 °C for 15 min. The samples were run by electrophoresis on the 3130 Genetic Analyzer (Applied Biosystems). The subsequent data were analyzed with GeneScan software and GeneScan-120 LIZ size standard.

**Table 4:** The oligonucleotide PCR primers used for amplification of genomic DNA to obtain template

Multiplex pool	Fragment name	Sequence 5′ → 3′	Length (bases)	Product size (bp)	SNV
1	P1PCR1	agcccggtaacctgtcgt	18	162	rs1801252
		ccatcagcagacccatgc	18		
	P1PCR2	tggaggaggtcaggcttaca	20	342	rs16947
		ggtgcagaattggaggtcat	20		rs5030867
					rs28371725
	P1PCR3	gtgtggtggcattgaggact	20	332	rs72549346
		gtggggacgcatgtctgt	18		
	P1PCR4	gatgcactggtccaaccttt	20	223	rs35742686
		ctggtgtaggtgctgaatgc	20		rs5030656
					rs72549353
					rs72549351
2	P2PCR1	gccttcaaccccatcatcta	20	328	rs1801253
		ggccctacaccttggattc	19		
	P2PCR2	ctcacctggtcgaagcagta	20	145	rs1065852
		ccatcttcctgctcctggt	19		rs5030862
	P2PCR3	cagctcggactacggtcatc	20	272	rs28371706
		cttgacaagaggccctgacc	20		
	P2PCR4	gtcctttcccaaacccatct	20	562	rs3892097
		gtggggctaatgccttcat	19		rs5030655
					rs5030865
					rs72549354
					rs72549356
					rs1135822

**Table 5:** The oligonucleotide primers for SNaPshot primer extension reactions

Multiplex pool	SNV	Primer direction	Peak to SNV correspondence	Primer length	Primer sequence (5′ → 3′)
1	rs28371725C/T	F(C/T)	C=C T=T	16	CCCCGCCTGTACCCTT
	rs1801252A/G	F(A/G)	A=A G=G	18	GACTCTCCCGCCAGCGAA
	rs72549346-/AC	R(T/G)	T=AC C=-	30	GACTGACTGACTGCCGTGATTCATGAGGTG
	rs16947A/G	F(A/G)	A=A G=G	40	GACTGACTGACTGACTGACTGAGGTCAGCCACCACTATGC
	rs5030656-/CTT	F(T/C)	T=CTT C=-	40	GACTGACTGACTGACTGACTGATGGCAGCCACTCTCACCT
	rs72549351-/AGTC	R(C/G)	C=AGTC G=-	46	GACTGACTGACTGACTGACTGACTGACTGACCCCCCCGAGACCTGA
	rs72549353-/AGTT	F(T/A)	T=AGTT A=-	46	GACTGACTGACTGACTGACTGACTGACCAGGTCATCCTGTGCTCAG
	rs35742686-/T	F(T/G)	T=T G=-	52	GACTGACTGACTGACTGACTGACTGACTGACTGACTGGGTCCCAGGTCATCC
	rs5030867T/G	R(A/C)	A=T C=G	52	GACTGACTGACTGACTGACTGACTGACTGACTCCTCCTGCTCATGATCCTAC
2	rs1801253C/G	F(C/G)	C=C G=G	15	CGCAAGGCCTTCCAG
	rs72549356-/GGGGCGAAAGGGGCGAAA	R(T/A)	T=GGGGCGAAAGGGGCGAAA A=-	18	GACTGCCCCTTTCGCCCC
	rs1065852G/A	R(C/T)	C=G T=A	36	GACTGACTGACTGACTGACTCTGGGCTGCACGCTAC
	rs3892097T/C	R(A/G)	A=T G=C	36	GACTGACTGACTGACTGACTGCATCTCCCACCCCCA
	rs28371706G/A	R(C/T)	C=G T=A	43	GACTGACTGACTGACTGACTGACTGACTCGCCTGTGCCCATCA
	rs5030862T/C	R(A/G)	A=T G=C	43	GACTGACTGACTGACTGACTGACTGACTCCCCTGCCACTGCCC
	rs72549354-/C	F(T/C)	T=- C=C	47	GACTGACTGACTGACTGACTGACTGACTCGACTCCTCCTTCAGTCCC
	rs5030655-/A	R(T/G)	T=T G=-	51	GACTGACTGACTGACTGACTGACTGACTGACTCAAGAAGTCGCTGGAGCAG
	rs1135822A/T	F(A/T)	A=A T=T	55	GACTGACTGACTGACTGACTGACTGACTGACTGACTGACTCATAGCGCGCCAGGA
	rs5030865A/C/T	F(A/C/T)	A=A C=C T=T	59	TGACTGACTGACTGACTGACTGACTGACTGACTGACTGACTCTTCTGCCCATCACCCAC

### CYP2D6 multiple-copy allele determination

The SNaPshot Multiplex System (Applied Biosystems) is a primer extension-based method developed for the analysis of SNVs. Theoretically, with the same SNV peak in the same reacting system with the same reaction conditions, a comparatively higher density means more DNA copies, and the ratios of any two peak densities of the same sample are relatively inflexible. We identified the copy numbers and duplicated/multiplicated allele by the ratio of the peak densities. In each pool, we designed one primer to identify an *ADRB1* gene’s SNV. *ADRB1* is a single-copy gene which allowed us to use the peaks of ADRB1 gene’s SNVs as the inner standard peaks. We calculated the ratios of the peak density of *CYP2D6* SNVs to the peak density of the *ADRB1* SNV. We set the reliability value range of the ratios of each SNV based on the values of the ratio of single-copy control samples with the same SNVs. If a ratio of a SNV of a known duplicated sample was greater than the maximum of the reference reliability range, this SNV was designated a duplication. We identified the copy number based upon that value. Based on published results and the values of the ratios, we assume all duplicated samples have the total copy number of three since this was the highest number of copy number variants (CNVs) in our controls.

### Activity scoring and predicted phenotype assignment

Each identified *CYP2D6* SNV was assigned a predicted enzyme activity score [1, 6]. Gene deletions were designated as an activity score of zero. The predicted enzyme phenotype was determined by addition of the individual gene activity scores, accounting for gene copies yielding decreased enzyme activity and gene duplications in each patient. A score of 0 was predicted to be a PM, 0.5 was predicted to be IM, 1–2 was predicted to be an EM, and 2.5 or greater was predicted to have a UM phenotype.

### Assay verification

Genotypes were confirmed with known reference genotype samples from 5 PMs, 4 IMs, and 24 EMs [8, 24]. Copy number variations were determined by Taqman Copy Number Assay (Life Technologies, CA) and then by pyrosequencing allele quantification in the known samples [24].

## Results

### Subjects

The demographics on the initial 79 subjects with unknown haplotypes in this cohort were as follows: the median age was 52 (IQR 45, 60), 46 (58.2 %) were males, 14 (19.9 %) were Hispanic/Latino, 37 (46.8 %) were Black or African American, 3 (3.8 %) were American Indian or Alaskan Native, 3 (3.8 %) were of Asian decent, 1 (1.3 %) was Native Hawaiian or Pacific Islander, and 38 (48.1 %) were Caucasian.

### Genotypes

Our genotyping method demonstrated consistent results with all 30 reference standards. The *CYP2D6* haplotype analysis revealed 7 (5.88 %) *CYP2D6* PMs, 18 (15.1 %) IMs, 89 (74.78 %) EMs, and 5 (4.2 %) UMs (Table 6). We also genotyped two variants of *ADRB1*, rs1801253 (Gly389Arg) and rs1801252 (Ser49Gly). The Gly389Arg genotype is Gly/Gly 18 (15.1 %), Gly/Arg 58 (48.7 %), and Arg/Arg 43 (36.1 %). The Ser49Gly genotype is Ser/Ser 82 (68.9 %), Ser/Gly 32 (26.9), and Gly/Gly 5 (4.2 %). See Table 7. *CYP2D6* allele frequencies are shown in Table 8. All genotypes were in the Hardy-Weinberg equilibrium (HWE) after allele designation by SNV identification and CNV determination.

### Copy number variants

We identified 13 subjects with CNVs with predicted enzyme activities scores ranging from 0 to 3 (Table 9). An example CNV determination is as follows. One sample had a genotype of *4/*41 and the total gene copy number of three. In this sample, we detected four heterozygous SNVs: rs1065852, rs3892097, rs28371725, and rs16947. The ratio of rs1065852 was 0.92, higher than the maximum of the reference reliability range (0.7–0.85). The ratio of rs3892097 was 0.98, higher than the maximum of the reference reliability range (0.6–0.9). The ratio of rs28371725 was 1.97, lower than the minimum of the reference reliability range (2.4–2.9). The ratio of rs16947 was 1.09, lower than minimum of the reference reliability range (1.28–1.38). All these ratios suggest that the variant allele, *4 was duplicated in this sample. Thus, the genotype is *4xN/*41. Since *4 is a non-functional allele, the activity score is assigned 0, and *41 is a reduced function allele, assigned an activity score of 0.5, even if the *4 is duplicated, the activity score remains 0.5. Hence, the genotype for this sample was designated IM (Table 6).

**Table 6:** Distribution of *CYP2D6* genotypes and phenotypes (*n* = 119, including 79 unknown subjects and 30 reference subjects)

Genotype	Number of subjects	Frequency (%)	Active score	Predicted phenotype	Phenotype frequency (%)
*1/*2xN	1	0.84	3.0	UM	4.2
*1xN/*2	1	0.84	3.0	UM	4.2
*1/*1xN	1	0.84	3.0	UM	4.2
*2/*2xN	1	0.84	3.0	UM	4.2
*1xN/*2	1	0.84	3.0	UM	4.2
*1/*1	15	12.61	2.0	EM	74.8
*1/*2	15	12.61	2.0	EM	74.8
*1/*3	4	3.36	1.0	EM	74.8
*1/*4	7	5.88	1.0	EM	74.8
*1/*6	2	1.68	1.0	EM	74.8
*1/*10	4	3.36	1.5	EM	74.8
*1/*17	8	6.72	1.5	EM	74.8
*1/*41	7	5.88	1.5	EM	74.8
*2/*2	4	3.36	2.0	EM	74.8
*2/*3	1	0.84	1.0	EM	74.8
*2/*4	2	1.68	1.0	EM	74.8
*2/*5	2	1.68	1.0	EM	74.8
*2/*10	2	1.68	1.5	EM	74.8
*2/*17	6	5.04	1.5	EM	74.8
*2/*40	1	0.84	1.0	EM	74.8
*2/*41	3	3.52	1.5	EM	74.8
*17/*17	1	0.84	1.0	EM	74.8
*1/*4xN	1	0.84	1.0	EM	74.8
*2xN/*12	1	0.84	2.0	EM	74.8
*2/*17xN	2	1.68	2.0	EM	74.8
*1/*5	1	0.84	1.0	EM	74.8
*4/*9	1	0.84	0.5	IM	16
*4/*17	2	1.68	0.5	IM	16
*5/*17	2	1.68	0.5	IM	16
*4/*41	4	3.36	0.5	IM	16
*5/*41	2	1.68	0.5	IM	16
*6/*17	1	0.84	0.5	IM	16
*6/*41	1	0.84	0.5	IM	16
*10/*40	1	0.84	0.5	IM	16
*17/*40	2	1.68	0.5	IM	16
*4xN/*10	1	0.84	0.5	IM	16
*4xN/*41	1	0.84	0.5	IM	16
*3/*4	1	0.84	0.0	PM	5.9
*4/*4	4	3.36	0.0	PM	5.9
*4xN/*5	2	1.68	0.0	PM	5.9

*PM* poor metabolizer, *IM* intermediate metabolizer, *EM* extensive metabolizer, *UM* ultra-rapid metabolizer

**Table 7:** *ADRB1* genotype

*ADRB1* haplotype	Number of subjects	Frequencies (%)	Expected metoprolol clinical effect
49Ser389Arg/49Ser389Arg	30	25.2	Threefold greater diastolic blood pressure reduction [7, 12]
49Ser389Arg/49Gly389Arg	8	6.7	Good responder [12]
49Ser389Gly/49Gly389Arg	22	18.5	Non-responders [7, 12]
49Ser389Gly/49Ser389Gly	16	13.4	Non-responders [7, 12]
49Ser389Gly/49Gly389Gly	2	1.7	Unknown, designated good responders by prior investigators [7, 12]
49Ser389Gly/49Ser389Arg	36	30.3	Good responder [12]
49Gly389Arg/49Gly389Arg	5	4.2	Unknown, designated good responders by prior investigators [7, 12]

**Table 8:** *CYP2D6* Allele frequencies

CYP2D6 allele	Number of subjects	Frequency (%)
*1	81	34
*1xN	3	1.3
*2	45	18.9
*2xN	3	1.3
*3	6	2.5
*4	25	10.5
*4xN	5	2.1
*5	9	3.8
*6	4	1.7
*9	1	0.4
*10	8	3.4
*12	1	0.4
*17	23	9.7
*17xN	2	0.8
*41	18	7.6 %

**Table 9:** Genotypes, activity score and predicted phenotypes for samples with gene duplications

Genotype (xN) before revision	Activity score	Predicted phenotype before revision	Genotype after revision	Activity score	Predicted phenotype after revision
*1/*2	3	UM	*1/*2xN	3	UM
*4/*41	0.5–1.0	IM OR EM	*4xN/*41	0.5	IM
*1/*2	3	UM	*1xN/*2	3	UM
*1/*1	3	UM	*1/*1xN^a^	3	UM
*1/*4	1.0–2.0	EM	*1/*4xN	1	EM
*4/*5	0	PM	*4xN/*5	0	PM
*2/*2	3	UM	*2/*2xN^a^	3	UM
*2/*17	2.0–2.5	UM OR EM	*2/*17xN	2	EM
*4/*10	0.5–1.0	IM	*4xN/*10^b^	0.5	IM
*1/*2	3	UM	*1xN/*2	3	UM
*2/*17	2.0–2.5	UM OR EM	*2/*17xN	2	EM
*4/*5	0	PM	*4xN/*5	0	PM
*2/*12	1.0–2.0	EM	*2xN/*12	2	EM

^a^The number of duplication alleles is far less than single-copy alleles. We assumed the subject was heterozygous for a single-copy allele and the duplicated/multiplicated allele

^b^No reference

*4 had a higher distribution or copy number in comparison with *10

### Assay verification

Genotyping results for the UM alleles showed a high degree of concordance between the Taqman Copy Number Assay paired with pyrosequencing quantification [24] and our SNaPshot methods. In fact, the only difference between the two assays was the identification of an additional CYP2D6*5 gene deletion with our multiplex longer-PCR method. Therefore, this assay is reliable for *CYP2D6* genotyping dependent upon the polymorphisms listed.

### Limitations

This method should be validated in an additional cohort with known genotypes to ensure concordance with other haplotype designations. This assay has not been validated to determine more than three CNVs because the control samples did not contain subjects with more than three. However, more than three CNVs of the identified SNVs are universally considered UMs. If additional CNVs of SNVs with associated lower activity scores are found, this may become important for haplotype distinction, depending upon the genotype at the second loci. Additional SNVs with altered predicted enzyme activity will not be captured unless the additional primers are added to the reaction pool. This flexibility of the method is an advantage though the assay is limited by 10 SNVs per pool and identification of allele duplications requires samples to be tested in batches in order to establish reference data. Oversaturation of the assay with additional primers in each pool may lead inconsistent detection of SNVs. Therefore, significantly increasing the number of SNVs identified will require larger volumes of DNA. With the presented assay, less than 60 ng of DNA was necessary to genotype 10 SNVs in these two genes in each sample.

## Discussion

We have demonstrated that the SNaPshot method of genotyping *CYP2D6* and *ADRB1* is reliable and efficient for rapidly identifying numerous SNVs. We demonstrate concordance with known reference standards using this method that requires no enzymatic digestion and can be performed at high volumes. The only difference was an additional identification of a CYP2D6*5 gene deletion not identified by the Taqman method. Less than 60-ng genomic DNA was used for each sample in our assay. The assay is flexible; it would be easy to add additional primers to cover more *CYP2D6* SNVs should this be necessary. This customizable assay has advantages given the speed of discovery of CYP SNV identification. As demonstrated by the success with *CYP2D6*, the multiplex SNaPshot method can be used for designing genotype assays for complex genotyping circumstances rapidly and inexpensively.

This method allows genotyping patients for two genes at only $40 per sample compared to commercially available microarray assays that cost in excess of $500 per sample. While the assay is limited by 10 SNVs per pool, this provided flexibility of the assay to add additional SNVs should they be clinically important. Additional SNVs would require additional DNA, but only 30 ng is required per pool allowing for significant up-scaling of the assay. This method is well suited for pharmacogenes with well-established clinical associations and a finite number of SNVs.

Subjects in our cohort had predicted phenotype frequencies similar to European populations.

Genotypes for *CYP2D6* and *ADRB1* demonstrate that 25 % may have a gene-gene interaction affecting their metoprolol therapy. Results in our cohort demonstrate a non-responder haplotype in 31.9 % of subjects. Liu et al. demonstrated a non-responder phenotype based upon these same haplotypes in 45.9 % of subjects in that trial [18]. Variability in ethnic proportions may explain this discrepancy though the frequencies are similar. These genes do not co-segregate, requiring knowledge of both genotypes to determine this interaction potential. Paired with clinical outcomes, these gene-gene interactions can be further clarified as the cohort matures. Overall, the multiplex SNaPshot represents a valuable tool for systems biology studies in need of flexible genotyping methods.

## Conclusion

Our multiplex SNaPshot protocol allows for specific and accurate detection of *CYP2D6* and *ADRB1* genotypes. This platform is flexible, simple, efficient, and suited for high throughput.
